# Healthcare provider’s perceptions of family values when making end-of-life care decisions for their children: developing and implementing a survey tool

**DOI:** 10.1177/26323524261467433

**Published:** 2026-07-09

**Authors:** Nithya Sivakumar, Stephanie Kukora, Cherie Ginwalla, Satyan Lakshminrusimha, Jennifer Rosenthal

**Affiliations:** 1 School of Medicine, 70083 University of California Davis, Sacramento, CA, USA; 2 Division of Neonatology and Bioethics Center, 4204 Children’s Mercy Kansas City, Kansas, MO, USA; 3 Department of Pediatrics, 70083 University of California Davis, Sacramento, CA, USA; 4 Division of Neonatology, Department of Pediatrics, University of California Davis, Sacramento, CA, USA

**Keywords:** end-of-life care, neonatal ICU, pediatric ICU, questionnaire design

## Abstract

**Background:**

Healthcare providers help families navigate end-of-life care decisions in the neonatal intensive care unit (NICU) and pediatric intensive care unit (PICU); however, limited studies exist regarding what values providers perceive families utilize when in these situations.

**Objective:**

To evaluate providers’ perceptions of values that families considered when making end-of-life care decisions for their child.

**Design:**

Develop a novel survey tool to understand providers’ perceptions of values families considered when making end-of-life care decisions for their children and distribute the survey tool to providers in a single quaternary care children’s hospital within a university hospital.

**Methods:**

The novel survey tool was composed of survey questions based on existing literature, revised based on expert feedback, then iteratively refined through cognitive interviews. Preliminary data from this survey tool was collected from eligible participants at one study site.

**Results:**

Feedback from nine experts and six cognitive interviews resulted in a 21-item survey. Ninety-five participants completed the survey. Participants reported that the relationship between the family and child, the child’s quality of life, and the child’s prognosis or disease course were most important to the family. Conversely, finances and prior experiences were ranked least important.

**Conclusions:**

A novel survey tool was created to identify the core values that families were perceived to favor by healthcare providers when making end-of-life care decisions for their children, as a means to improve end-of-life care discussions at the bedside. Pilot data suggests that providers may understand the values that families utilize, though may not understand *how* they prioritize them. Future studies comparing family and provider responses, with mirrored surveys, are necessary to create decision support tools and to improve provider education in end-of-life care decision-making.

## Introduction

End-of-life care decision-making in the neonatal intensive care unit (NICU) and pediatric intensive care unit (PICU) is complex. These decisions and surrounding discussions involve exploration into what the family considers important for their loved one, in the context of their prognosis and disease course, consideration of what would be in the perceived best interests of the child, and overarching guidance by the values of the family, with important input from the child’s healthcare team.^1–4^

However, the end-of-life care discussions occurring in the NICU and PICU may not adequately center the information families need to make these complex decisions. Pediatric healthcare providers have been noted to focus on possible outcomes, including the probability of morbidity and death, when facilitating end-of-life care discussions with families, and emphasize spirituality, emotions, and hope less frequently, although families often desire further communication on the latter aspects.^5,6^ The cause of this discrepancy is unclear, though may stem from provider discomfort with discussing unfamiliar aspects of spirituality and family values, believing their primary role is to communicate factual information only, or prognostic uncertainty—nevertheless, this primary focus on data may affect the end-of-life care decisions made.^6–10^

There are few recently published models for shared decision-making in pediatric palliative care,^11^ but its wide-spread utilization by non-palliative pediatric providers remains seemingly limited. Pediatric palliative care providers are uniquely equipped to navigate difficult conversations and help supply families with critical information, however, pediatric providers, including pediatric clinicians, advance practice care providers, residents, fellows, nurses and social workers, often do not receive the same training,^12,13^ leading to a perpetuation of the disparity between the information that families hope to receive and the information communicated to them. Moreover, the need for pediatric providers to navigate difficult conversations without appropriate training can exacerbate the moral distress that providers already experience, distress that further negatively affects end-of-life care decision-making.^14,15^

Understanding the moral and practical values families employ when making complex decisions in the NICU and PICU will improve the effectiveness of healthcare providers navigating these discussions. End-of-life care decision-making is an emotionally vulnerable time; a provider-reported survey tool to gather perceptions of what families prioritize when making end-of-life care decisions for their children in the NICU and PICU collects critical preliminary data without causing undue harm to grieving families. Ultimately, the data collected in this study and in subsequent, larger trials would be beneficial to guide provider education. We thus aimed to create and pilot a survey tool for healthcare providers to assess considerations that families were perceived to value when making end-of-life care decisions for their child.

## Methods

This is a cross-sectional survey study of provider-reported perceptions of family values and considerations when making end-of-life care decisions for their child. This study contained two components: (1) development of the research survey and (2) preliminary survey data collection at a single center. The survey tool was developed to fulfill the objective of collecting healthcare providers’ perceptions of what families value and prioritize when making end-of-life care decisions for their children. The University of California, Davis Institutional Review Board approved this study.

### Setting

This study was conducted at a 121-bed metropolitan quaternary care children’s hospital within a university hospital. This hospital serves as a referral center for children across a 33-county region spanning 65,000 square miles and routinely receives pediatric transfers from 30 hospitals in the region. The level IV NICU has 49 beds and admits over 900 neonatal patients annually. The PICU has 24 beds (including cardiac, surgical, and extracorporeal membrane oxygenation patients) and treats over 1,600 children annually.

### Eligibility criteria

Participants for both stages of this study– research survey development and survey data collection – were considered eligible to participate if they were an attending physician, fellow, resident, advanced care provider (nurse practitioner or physician assistant), nurse, or social worker working in the NICU or PICU. Additionally, eligible participants were required to have worked with a patient who required life sustaining therapy and been a party in end-of-life care discussions with the patient’s family within the past two years. As this survey was designed in English, only participants with English language preference were eligible to participate.

### Research survey development

#### Preliminary survey development

The preliminary survey was developed de novo using existing literature^5,16–19^ and designed to be used to assess perceptions of family values only. Though clinical providers’ experiences notably contribute to end-of-life care decision-making, questions regarding clinician’s experiences were not included in the research survey, but rather, collected alongside demographic information. We identified five relevant domains based on the literature: (1) provider input and involvement in end-of-life care decision-making, (2) religion or cultural values, (3) prognosis, disease course, quality of life and developmental outcomes, (4) finances, and (5) the child’s role in the family. The preliminary survey contained 29-items and utilized a 4-point response scale, from “strongly agree” to “strongly disagree”.

#### Content expert assessment

The preliminary survey was electronically distributed to 15 content experts in the field, with nine (60%) responses received. These nine experts represented 8 distinct hospitals. Each content expert was asked to rate each survey question’s relevance to assessing family values when making decisions at the end-of-life for their children, to identify any missing or duplicative questions, and to comment on the clarity or wording of each question.

Six content experts rated each question on a 4-point scale, from “not relevant” to “highly relevant”. Responses were used to compute the item-level validity index, as described by Lynn.^20^ If the item-level validity index of a question did not meet the minimum threshold, then the question was revised or removed from the survey. An additional three content experts provided feedback but did not rate the survey questions. Their feedback was taken into consideration in conjunction with item-level content validity index scores to help decide if questions should be removed, revised, or saved in the second iteration of the survey.

#### Cognitive interviews

After feedback from content experts was incorporated into the survey, one-on-one cognitive interviews were held with eligible participants between January 2024 and May 2024. Recruitment was completed through email outreach to role- and department-specific email listservs, and participants were instructed to email one investigator (NS) regarding their interest in participating. Participants engaged in a one-hour session that included completing the survey using the think-aloud technique, followed immediately by a semi-structed interview to assess comprehension and usability using probing techniques.^21,22^ Verbal consent for participation in the cognitive interview was obtained and participants received a $10 gift card for their participation.

Cognitive interviews were completed by one researcher (NS), after training completed with a study team member with known experience (JLR), via videoconference with audio recording to aid in transcript creation. The transcripts of each interview were summarized and used to code interviews thematically. After completing three interviews, three researchers (NS, CG, JLR) met to review and discuss the summaries and codes. With consensus agreement, the group added, modified, or removed questions to improve comprehension and usability of the survey tool. This process was repeated until no further areas were identified for improvement.^21,22^

### Survey data collection

#### Recruitment and data collection

Potential participants were invited by email through title- and department-related listservs to complete the survey between May 2024 and November 2024. Additional recruitment was conducted through IRB-approved paper advertisements displayed in designated provider spaces in the NICU and PICU. The survey was only distributed electronically.

Every participant was required to complete a pre-screening tool, built into the survey to determine eligibility, prior to moving to the final research survey. All other components of the survey were optional. Upon completion, each participant was offered a $10 gift card.

#### Data analysis

Data were analyzed using descriptive statistics and two sample t-tests. Demographics and case-specific questions were analyzed using chi-square testing and descriptive statistics. Responses to the question that asked participants to rank values by what they perceived was most important to families were compared between NICU- and PICU- based participants using two sample t-tests assuming equal variances; overall mean, standard deviation, and p-values were also reported for each question. Data from questions that asked participants to indicate on a Likert scale how strongly they agreed with statements regarding family’s values and decision-making were described using median, first, and third quartiles.

## Results

### Research survey development

#### Content expert assessment

The preliminary survey contained 29 questions, 14 of which were removed or altered given an item-level content validity index of less than 0.83^20^. Of these 14 questions, 6 were removed and 8 were amended. Suggestions regarding the clarity of questions were incorporated. All but one free response question was removed. The resulting instrument had 22 questions, with one additional optional free response. Twenty of these questions utilized a 5-point scale from “strongly disagree” to “strongly agree”, one question was a binary “yes” or “no” question, and one asked participants to rank values in order from most to least important in the family’s end-of-life care decision-making processes.

#### Cognitive interviews

Six interviews were conducted over videoconference between April and May of 2024. The 20-question survey was revised within two rounds, after which there were no other issues identified by the participants. Participants reported concerns with the response scale, with question clarity, and with missing items. All questions were amended to fit the 5-point scale previously described; five questions were edited to impart clarity, two questions were added, and two questions were combined into one for a final survey with 21 questions and one free response.

#### Final survey

**Figure 1.**
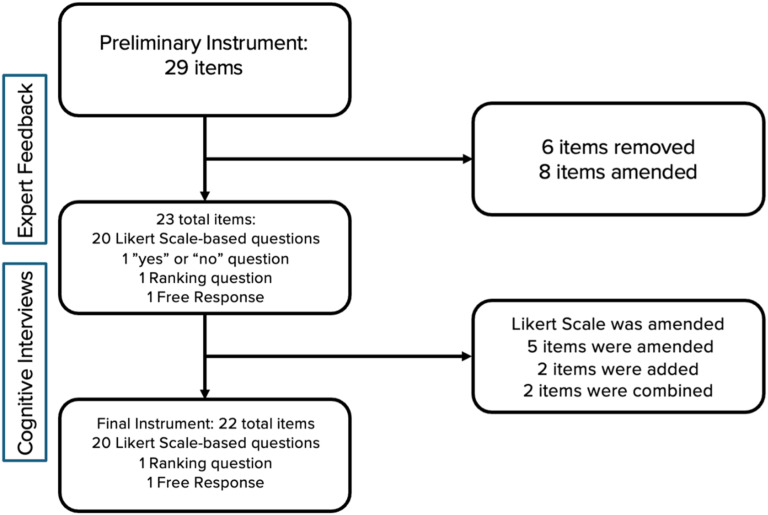
Flow diagram of the development of the survey tool.

The final research survey included 20 Likert scale-based questions, one ranking question, and one free response question (Figure 1, Supplementary File 1). Every participant was invited to complete a screening tool to assess eligibility, and if eligible, were allowed to move to the final research survey. The final research survey had each participant consider their most recent experience when taking part in end-of-life care decision-making with families for their children, and answer each question based on that experience. At the end of the survey, we queried participants’ demographics, position and experience, specifics pertinent to the case participant’s envisioned while responding to the survey, their personal level of agreement with decisions made by the family, and their personal experience of burnout to contextualize the responses received.

### Survey data collection

#### Survey results

Among the 483 healthcare providers invited to complete the survey, 95 participants completed a portion of the survey with missingness ranging from 3 - 82% of survey questions; 73 (76.8%) completed the entire survey with no missingness and were included in the final analysis. We had a 19.7% response rate, with 15.1% completing the research survey and 14.3% completing the entire survey.

Seventy-three completed surveys were included in the final analysis: 40 (54.8%) participants were from the NICU, and 29 (39.7%) were from the PICU; four (5.5%) did not disclose which unit they worked in. The roles of the participants are listed in Table 1.

**Table 1.** Demographic information for respondents and case-specific information.

​	NICU (40)	PICU (29)	All (69)
Profession			
*Attending*	4	8	12
*Advanced Care Provider (PA/NP)*	5	2	7
*Nurse*	26	14	40
*Resident*	2	3	5
*Social Work*	3	1	4
*Fellow*	0	1	1
Provider agreement with the family’s decision to continue or withdraw care			
*Strongly Agree*	18	10	28
*Agree*	10	8	18
*Neither*	5	5	10
*Disagree*	4	6	10
*Strongly Disagree*	3	0	3
Provider agreement with the process the families used to decide whether to continue or withdraw care			
*Strongly Agree*	15	9	24
*Agree*	11	12	23
*Neither*	9	3	12
*Disagree*	4	5	9
*Strongly Disagree*	1	0	1
Patients hospitalized for the entirety of their life	40	3	43
Families aware of disease/condition prior to hospitalization	18	8	26
Approximate length of time between hospitalization and discussions of end-of-life care			
*Within hours*	9	4	13
*Within days*	15	9	24
*Within weeks*	11	15	26
*Within months*	5	1	6
Provider’s perceptions of the certainty of the patient’s prognosis			
*Strongly certain*	23	16	39
*Somewhat certain*	13	7	20
*Neither certain nor uncertain*	4	4	8
*Somewhat uncertain*	0	2	2
*Strongly uncertain*	0	0	0
Patients for whom life sustaining interventions were continued	15	12	27

Most respondents agreed with the decision the family made (67%) and the process by which they made the decision (68%). The cases respondents considered differed in terms of parents’ knowledge of the condition prior to hospitalization and length of stay, though the majority reported that the prognosis was certain at the time decision-making occurred (Table 1). Fifty-two (75.4%) respondents reported experiencing some level of moral distress in caring for their patient, with 9 (13%) reporting that they experienced “significant” moral distress.

Results of perceptions of which factors were considered by parents and how these were prioritized by parents are listed in Table 2 and Table 3, respectively. Overall, respondents agreed that the families considered their child’s likelihood of survival, likelihood of permanent disability, disease course, bond with their child, pain and non-pain symptoms, as well as present and possible future quality of life. Additionally, respondents perceived that families considered their religious and cultural beliefs when making end-of-life care decisions for their children (Table 2). When asked to rank the values that families were perceived to prioritize, respondents reported that the relationship between the family and the child, the child’s quality of life, and the child’s prognosis or disease course were the most important—conversely, finances and prior experiences with decision-making were ranked as the least important (Table 3). There were no significant differences between values providers perceived to be important to families in the NICU and PICU, however, this pilot study was not designed to detect significant differences.

**Table 2.** Research survey results from questions with answers on a likert scale from “Strongly Disagree” to “Strongly Agree”, reported as median (first interquartile, third interquartile).

​	All	NICU	PICU	N/A
Q1: The family requested the care team’s opinion when deciding end of life care for their child.	4 (3,4)	4 (3,5)	4 (3,4)	4 (4,4)
Q2: The family made decisions that were consistent with the team’s recommendations.	4 (3,4)	4 (2.75,4)	3 (3,4)	4 (3.75, 4)
Q3: The family was provided with adequate opportunities to share their hopes with the care team.	4 (4,5)	4 (4,5)	4 (4,5)	4.5 (3.5, 4.25)
Q4: The family was provided with adequate opportunities to share their fears with the care team.	4 (4,5)	4 (4,5)	4 (4,5)	4 (3.5, 4.25)
Q5: Family members had adequate emotional preparation from the care team.	4 (3,4)	4 (3,4)	4 (4,4)	2.5 (2, 3.5)
Q6: Family members had adequate emotional support from the care team.	4 (4,5)	4 (4,5)	4 (4,4)	4 (3,5)
Q7: The family’s religion or spirituality influenced the decisions made for their child.	4 (4,5)	4 (4,5)	4 (3,5)	3.5 (3, 4.25)
Q8: The family’s cultural beliefs influenced the decisions made for their child	4 (4,5)	4 (4,5)	4 (3,5)	4 (3.75, 4.25)
Q9: The family had prior experience(s) with end-of-life care decision-making.	2 (1,3)	2 (1,2)	2 (2,3)	2.5 (1.75, 3)
Q10: Their child’s likelihood of survival influenced the decisions made for their child.	4 (3,5)	4 (2,4)	4 (3,5)	4 (3.5, 4)
Q11: Their child’s possibility of permanent disability influenced the decisions made for their child.	4 (2,5)	4 (2,5)	4 (2,4)	3 (2,4)
Q12: Their child’s present and future disease course influenced the decisions made for their child.	4 (3,5)	4 (2.75, 5)	4 (3,4)	4 (3.5,4)
Q13: The family’s finances influenced the decisions made for their child.	2 (1,3)	2 (1,3)	2 (2,3)	2 (1.75,2)
Q14: The family considered the bond with their child when making decisions for them.	4 (4,5)	4 (4,5)	4 (3,5)	4 (4,4)
Q15: The family considered their competing responsibilities when making decisions for their child.	3 (2,4)	3 (2,4)	3 (2,3)	3 (2.75, 3.25)
Q16: Their child’s pain influenced the decisions made for their child.	4 (3,5)	4 (3,5)	4 (3,5)	4 (3.5, 4)
Q17: Their child’s non-pain symptoms influenced the decisions made for their child.	4 (3,4)	4 (3,4)	3 (3,4)	4 (3.5, 4)
Q18: Their child’s current quality of life influenced the decisions made for their child.	4 (3,4)	4 (3,5)	4 (3,4)	4 (3.75, 4)
Q19: Their child’s future quality of life influenced the decisions made for their child.	4 (3,5)	4 (4,5)	4 (3,4)	4 (3.5, 4.25)
Q20: The family’s quality of life influenced decisions made.	3 (2,4)	3 (2,4)	3 (2,4)	2.5 (2, 3.25)

**Table 3.** Research survey result from ranking question. Respondents were asked to rank values that their family considered from most (1) to least (10) important, reported as average (standard deviation).

​	All	NICU	PICU	p-value
Relationship between family and child	3.4 (1.91)	3.7 (2.00)	2.9 (1.81)	0.11
Relationship between family and care team	6.4 (2.30)	6.1 (2.30)	6.8 (2.19)	0.19
Religious/Spiritual identity	4.5 (2.56)	4.7 (2.58)	4.5 (2.67)	0.79
Cultural beliefs	4.9 (2.44)	5.1 (2.62)	4.8 (2.29)	0.64
Quality of life for their child	3.0 (2.11)	3 (2.14)	3.1 (2.12)	0.72
Quality of life for their family	6.2 (1.89)	6.2 (1.97)	5.9 (1.85)	0.53
Likelihood of permanent disability	5.2 (2.17)	5 (2.12)	5.4 (2.31)	0.53
Prognosis/Disease course	3.6 (2.13)	3.5 (2.14)	3.9 (2.22)	0.37
Finances	9.2 (0.90)	9 (0.97)	9.3 (0.84)	0.37
Prior experiences with end-of-life care decision-making	8.6 (1.95)	8.7 (1.87)	8.3 (2.15)	0.39

## Discussion

We sought to understand healthcare provider’s perceptions of family values and priorities during end-of-life care decision-making for their children in the NICU and PICU, and we developed a novel survey tool towards that purpose. Our pilot revealed that this survey can be completed with low missing values.

The survey was intended as a first step towards understanding the priorities families have when making end-of-life care decisions for their children to improve and inform end-of-life care discussions at the bedside. While our study focused solely on the perspectives of healthcare providers, true understanding of family values, how they contribute to end-of-life decision-making, and the differences between healthcare providers’ and families’ perceptions and families’ values requires surveying both families and members of the care team. The scope of this study was to develop a survey tool to assess provider perceptions and additionally intended to serve as a starting point for developing similar survey tools that would be appropriate to ascertain families’ perspectives.

Study participants in the NICU and PICU identified that families value current and future quality of life for their child, religious and cultural factors, pain and non-pain symptoms, the relationship between the family and child, competing responsibilities of the family, likelihood of permanent disability or death, and prognosis and disease course, which is consistent with what has been reported in the literature by parents.^5,16,19,23–25^ When respondents were asked to rank perceived values from most to least important, they reported prognosis and disease course, quality of life, and relationship between the family and child to be most important; however, bereaved parents of children who died in the PICU have previously reported quality of life, likelihood of clinical improvement, likelihood of death, and perceived pain to be the most salient factors for end-of-life care decision-making for their children.^19^ Additionally, respondents perceived past experiences of or previous involvement in end-of-life care decision-making to be of less value for families making similar decisions for their children; however, in a prospective study of 14 parents of children in the PICU, past experiences with end-of-life heavily influenced parents’ decision-making, noting that parent’s often compared their child’s situation with that of loved ones who have previously passed away.^23^ Respondents viewed finances to not play a significant role in end-of-life care decision-making—consistent with prior research^19^—though whether finances play a significant role in decision-making is difficult to assess, as finances may not be routinely brought up in family meetings. The discrepancies elicited by our survey item requires further investigation—if reproduced in larger studies and the difference corroborated with a mirrored, family-focused survey tool, it highlights a gap between true family values and provider perceptions of the same, and indicate a need for education interventions so that providers are better equipped to delve into what values families prioritize most when discussing end-of-life care for their children.

Our survey did not discern a difference between responses from respondents in the NICU and PICU, although there is emerging evidence that end-of-life care decision-making differs between units.^26–29^ At our hospital, neonates requiring advanced cardiac support, including extracorporeal membrane oxygenation, are transferred to the PICU where our cardiac intensive care subunit is located, contributing to our inability to discern a difference.

Most respondents (75.4%) from the NICU and PICU reported that they experienced some level of moral distress in caring for their pediatric patient. End-of-life care decision-making in pediatrics is emotionally fraught, with clinical providers often experiencing moral distress rooted in value differences.^15,30,31^ The experience of moral distress, especially when value conflicts exist, can often be exhibited as rude or dismissive behaviors that risk the parent-provider relationship.^32^ Although differences in values will persist, providers’ acknowledgement of specific family values in end-of-life care decision-making may allow for better coping and reduced moral distress in the NICU and PICU.^30^

There are several limitations to this study. This study asked participants to consider the most recent patient where they were involved in the end-of-life care discussions with their family. Given the limited number of patients that required end-of-life care discussions in the NICU and PICU at our hospital during our study period, multiple providers may have envisioned the same case, possibly skewing results. This may be mitigated in larger trials, but unavoidable in this study. Additionally, we had a low response rate, likely secondary to our methods of outreach—our survey was primarily distributed through email listservs, and secondarily through paper flyers in teamwork spaces, methods that can easily be overlooked in high-stakes clinical settings. Targeted emails to potential participants, rather than emails distributed through listservs, and in-person recruitment efforts may increase response rate. While this survey was developed and pilot tested at a single site, larger studies in a variety of children’s hospital settings (including free-standing, university-affiliated, and community children’s hospitals) should be completed to confirm our study findings.

Despite these limitations, the results of our novel survey reveal interesting preliminary results, that, if corroborated by future studies in larger sample sizes with a mirrored, family-focused survey, may serve to guide clinical trainee and provider education in end-of-life care decision-making. The preliminary data collected shows that while providers may demonstrate understanding of the core values that families prioritize when making end-of-life care decisions, there may be differences in what providers believe is most important to families when compared to published literature. By constructing support tools and education for providers to elicit values that families prioritize, and *how* they prioritize them, we encourage end-of-life care discussions that support the needs of the family, positively influence the patient’s and families’ experience during their hospitalization,^24,33–35^ as well as serve to mitigate moral distress as experienced by providers.^30^

## Supplemental material

Supplemental material - Healthcare provider’s perceptions of family values when making end-of-life care decisions for their children: Developing and implementing a survey tool Supplemental material for Healthcare provider’s perceptions of family values when making end-of-life care decisions for their children: Developing and implementing a survey tool by Nithya Sivakumar, Stephanie Kukora, Cherie Ginwalla, Satyan Lakshminrusimha, and Jennifer Rosenthal in Palliative Care and Social Practice.

## Data Availability

The final version of the survey utilized is available as a supplemental file in this article; demographic and supplemental questions, as well as preliminary data collected, is available by contacting the corresponding author.*

## References

[1] Committee on Hospital Care. American Academy of Pediatrics . Family-centered care and the pediatrician’s role. Pediatrics 2003; 112: 691–697. 12949306

[2] Boland L Graham ID Légaré F , et al. Barriers and facilitators of pediatric shared decision-making: a systematic review. Implement Sci IS 2019; 14: 7. 10.1186/s13012-018-0851-5 30658670 PMC6339273

[3] Weiss EM Xie D Cook N , et al. Characteristics Associated With Preferences for Parent-Centered Decision Making in Neonatal Intensive Care. JAMA Pediatr 2018; 172: 461–468. 10.1001/jamapediatrics.2017.5776 29554176 PMC5875325

[4] Pellikka H-K Axelin A Sankilampi U , et al. Shared responsibility for decision-making in NICU: A scoping review. Nurs Ethics 2023; 30: 462–476. 10.1177/09697330221134948 36688269 PMC10185855

[5] Boss RD Hutton N Sulpar LJ , et al. Values Parents Apply to Decision-Making Regarding Delivery Room Resuscitation for High-Risk Newborns. Pediatrics 2008; 122: 583–589. 10.1542/peds.2007-1972 18762529

[6] Bastek TK Richardson DK Zupancic JAF , et al. Prenatal consultation practices at the border of viability: a regional survey. Pediatrics 2005; 116: 407–413. 10.1542/peds.2004-1427 16061596

[7] Editors TPlM . Making the “Right” Health Care Decisions: Why Values Matter. PLOS Med 2009; 6: e1000136. 19707576 10.1371/journal.pmed.1000136 PMC2725303

[8] Janvier A Barrington K Farlow B . Communication with parents concerning withholding or withdrawing of life-sustaining interventions in neonatology. Semin Perinatol 2014; 38: 38–46. 10.1053/j.semperi.2013.07.007 24468568

[9] Needle JS Mularski RA Nguyen T , et al. Influence of personal preferences for life-sustaining treatment on medical decision making among pediatric intensivists. Crit Care Med 2012; 40: 2464–2469. 10.1097/CCM.0b013e318255d85b 22809913

[10] Kaemingk BD Carroll K Thorvilson MJ , et al. Uncertainty at the Limits of Viability: A Qualitative Study of Antenatal Consultations. Pediatrics 2021; 147: e20201865. 10.1542/peds.2020-1865 33658319

[11] Cai S Cheng L Wang R , et al. A shared decision-making model in pediatric palliative care: a qualitative study of healthcare providers. BMC Palliat Care 2023; 22: 190. 10.1186/s12904-023-01307-0 38012611 PMC10683132

[12] Boss RD Hutton N Donohue PK , et al. Neonatologist Training to Guide Family Decision Making for Critically Ill Infants. Arch Pediatr Adolesc Med 2009; 163: 783–788. 10.1001/archpediatrics.2009.155 19736330 PMC2843907

[13] Kolarik RC Walker G Arnold RM . Pediatric resident education in palliative care: a needs assessment. Pediatrics 2006; 117: 1949–1954. 10.1542/peds.2005-1111 16740835

[14] Jeong S Knackstedt A Linebarger JS , et al. Moral Distress and Pediatric Palliative Care. Children 2024; 11: 751. 10.3390/children11070751 39062203 PMC11274977

[15] Prentice T Janvier A Gillam L , et al. Moral distress within neonatal and paediatric intensive care units: a systematic review. Archives of Disease in Childhood 101: 701–708. 2016. 10.1136/archdischild-2015-309410 26801075

[16] Kukora SK Boss RD . Values-based shared decision-making in the antenatal period. Semin Fetal Neonatal Med 2018; 23: 17–24. 10.1016/j.siny.2017.09.003 28917833

[17] Kirschbaum MS . Life Support Decisions for Children: What Do Parents Value? Adv Nurs Sci 1996; 19: 51–71. 10.1097/00012272-199609000-00007 8866000

[18] Xafis V Wilkinson D Sullivan J . What information do parents need when facing end-of-life decisions for their child? A meta-synthesis of parental feedback. BMC Palliat Care 2015; 14: 19. 10.1186/s12904-015-0024-0 25924893 PMC4424961

[19] Meyer EC Burns JP Griffith JL , et al. Parental perspectives on end-of-life care in the pediatric intensive care unit. Crit Care Med 2002; 30: 226–231. 10.1097/00003246-200201000-00032 11902266

[20] Lynn MR . Determination and Quantification Of Content Validity. Nurs Res 1986; 35: 382–386. 10.1097/00006199-198611000-00017 3640358

[21] DeVellis R . Scale Development: Theory and Applications, Fourth Edition. SAGE Publications.

[22] Willis G . Cognitive Interviewing: A tool for improving questionnaire design. SAGE Publications.

[23] Sharman M Meert KL Sarnaik AP . What influences parents’ decisions to limit or withdraw life support? Pediatr Crit Care Med 2005; 6: 513–518. 10.1097/01.pcc.0000170616.28175.d9 16148808

[24] Mitchell S Spry JL Hill E , et al. Parental experiences of end of life care decision-making for children with life-limiting conditions in the paediatric intensive care unit: a qualitative interview study. BMJ Open 2019; 9: e028548. 10.1136/bmjopen-2018-028548 PMC6528052 31072863

[25] Michelson KN Koogler T Sullivan C , et al. Parental Views on Withdrawing Life-Sustaining Therapies in Critically Ill Children. Arch Pediatr Adolesc Med 2009; 163: 986–992. 10.1001/archpediatrics.2009.180 19884588 PMC2873853

[26] Laventhal N Spelke MB Andrews B , et al. Ethics of resuscitation at different stages of life: a survey of perinatal physicians. Pediatrics 2011; 127: e1221–e1229. 10.1542/peds.2010-1031 21502232

[27] Fontana MS Farrell C Gauvin F , et al. Modes of death in pediatrics: differences in the ethical approach in neonatal and pediatric patients. J Pediatr 2013; 162: 1107–1111. 10.1016/j.jpeds.2012.12.008 23312685

[28] Janvier A Leblanc I Barrington KJ . Nobody likes premies: the relative value of patients’ lives. J Perinatol Off J Calif Perinat Assoc 2008; 28: 821–826. 10.1038/jp.2008.103 18633422

[29] Janvier A Leblanc I Barrington KJ . The best-interest standard is not applied for neonatal resuscitation decisions. Pediatrics 2008; 121: 963–969. 10.1542/peds.2007-1520 18450900

[30] Kukora S Laventhal N Keefer P , et al. Transgressing Moral Imperatives: Ethical Stress, Virtues and Values Conflict in Pediatric Death. Pediatric Ethicscope 2018; 31: 38–50. https://pediatricethicscope.org/article/transgressing-moral-imperatives/ (accessed 14 March 2025.

[31] Trotochaud K Coleman JR Krawiecki N , et al. Moral Distress in Pediatric Healthcare Providers. J Pediatr Nurs Nurs Care Child Fam 2015; 30: 908–914. 10.1016/j.pedn.2015.03.001 25869472

[32] Krenz C Spector-Bagdady K De Vries R , et al. Parents’ perspectives on values and values conflicts impacting shared decision making for critically ill neonates. J Peds Ethics 2024; 3: 46–59. 10.63292/fjcf5302

[33] Polakova K Ahmed F Vlckova K , et al. Parents’ experiences of being involved in medical decision-making for their child with a life-limiting condition: A systematic review with narrative synthesis. Palliat Med 2024; 38: 7–24. 10.1177/02692163231214414 38053373 PMC10798032

[34] Sullivan J Monagle P Gillam L . What parents want from doctors in end-of-life decision-making for children. Arch Dis Child 2014; 99: 216–220. 10.1136/archdischild-2013-304249 24311188

[35] Meert KL Eggly S Pollack M , et al. Parents’ perspectives on physician-parent communication near the time of a child’s death in the pediatric intensive care unit. Pediatr Crit Care Med J Soc Crit Care 2008; 9: 2–7. 10.1097/01.pcc.0000298644.13882.88 PMC3198033 18477906

