# Supplementary material for: Healthcare provider’s perceptions of family values when making end-of-life care decisions for their children: developing and implementing a survey tool
Source: Palliat Care Soc Pract. 2026 Jul 9;20:26323524261467433. doi: 10.1177/26323524261467433 (PMC13351224; doi:10.1177/26323524261467433)
Supplement: Supplemental material - Healthcare provider’s perceptions of family values when making end-of-life care decisions for their children: Developing and implementing a survey tool [file sj-pdf-1-pcr-10.1177_26323524261467433.pdf]

Supplementary File 1: Final research survey

Directions:
This survey asks questions about your perceptions of what the **family** values in the decision-making process between continuing life sustaining therapy (i.e. persistent use of mechanical ventilation, etc.) or withdrawing care for their child.

Please answer each question based on your **most recent** encounter where you, as a member of the care team, were working with a family to decide end-of-life care for their child.

How strongly do you agree with the following statements? Please select one answer choice for each statement below.
Answer Choices: Strongly Agree, Agree, Neither Agree nor Disagree, Disagree, Strongly Disagree

1. The family requested the care team's opinion when deciding end of life care for their child.
2. The family made decisions that were consistent with the team's recommendations.
3. The family was provided with adequate opportunities to share their hopes with the care team.
4. The family was provided with adequate opportunities to share their fears with the care team.
5. Family members had adequate emotional preparation from the care team.
6. Family members had adequate emotional support from the care team.
7. The family's religion or spirituality influenced the decisions made for their child.
8. The family's cultural beliefs influenced the decisions made for their child.
9. The family had prior experience(s) with end-of-life care decision making.
10. Their child's likelihood of survival influenced the decisions made for their child.
11. Their child's possibility of permanent disability influenced the decisions made for their child.
12. Their child's present and future disease course influenced the decisions made for their child.
13. The family's finances influenced the decisions made for their child.
14. The family considered the bond with their child when making decisions for them.
15. The family considered their competing responsibilities when making decisions for their child.
16. Their child's pain influenced the decisions made for their child.
17. Their child's non-pain symptoms influenced the decisions made for their child.
18. The child's current quality of life influenced decisions made.

19. The child's future quality of life influenced decisions made.

20. The family's quality of life influenced decisions made.

21. Please rank (from most to least important) the factors that you believe mattered for your patient's family when making end of life decisions for their child.
    a. Relationship between family and child
    b. Relationship between family and care team
    c. Religious Identity
    d. Cultural beliefs
    e. Finances
    f. Quality of life for their child.
    g. Quality of life for their family
    h. Prognosis/Disease Course
    i. Likelihood of permanent disability
    j. Prior experiences with end-of-life care decision making

22. Optional: Are there any other factors that came up in your conversations with the family as important for end-of-life care decision making? Please type your answer here:
